# Integration of Sentence-Level Semantic Information in Parafovea: Evidence from the RSVP-Flanker Paradigm

**DOI:** 10.1371/journal.pone.0139016

**Published:** 2015-09-29

**Authors:** Wenjia Zhang, Nan Li, Xiaoyue Wang, Suiping Wang

**Affiliations:** 1 Center for Studies of Psychological Application and School of Psychology, South China Normal University, Guangzhou, China; 2 Key Laboratory of Mental Health and Cognitive Science of Guangdong Province,South China Normal University, Guangzhou, China; Zhejiang Key Laborotory for Research in Assesment of Cognitive Impairments, CHINA

## Abstract

During text reading, the parafoveal word was usually presented between 2° and 5° from the point of fixation. Whether semantic information of parafoveal words can be processed during sentence reading is a critical and long-standing issue. Recently, studies using the RSVP-flanker paradigm have shown that the incongruent parafoveal word, presented as right flanker, elicited a more negative N400 compared with the congruent parafoveal word. This suggests that the semantic information of parafoveal words can be extracted and integrated during sentence reading, because the N400 effect is a classical index of semantic integration. However, as most previous studies did not control the word-pair congruency of the parafoveal and the foveal words that were presented in the critical triad, it is still unclear whether such integration happened at the sentence level or just at the word-pair level. The present study addressed this question by manipulating verbs in Chinese sentences to yield either a semantically congruent or semantically incongruent context for the critical noun. In particular, the interval between the critical nouns and verbs was controlled to be 4 or 5 characters. Thus, to detect the incongruence of the parafoveal noun, participants had to integrate it with the global sentential context. The results revealed that the N400 time-locked to the critical triads was more negative in incongruent than in congruent sentences, suggesting that parafoveal semantic information can be integrated at the sentence level during Chinese reading.

## Introduction

Reading of text requires readers to move their eyes to bring the material of interest into their foveal vision field, given that the information acquired during one fixation time is quite limited [1]. However, since the processing of a word starts when it is in the parafoveal vision, previous studies using different techniques investigated the processing of parafoveal words. Previous studies with eye movements technique were mostly concerned with what kind of information in the parafovea can be extracted during on-line reading. There is now considerable agreement that orthographic and phonological information can be obtained in the parafovea [2–5]. Furthermore, although studies using alphabetic languages have not consistently found that higher-level information, such as semantic information, could be extracted from parafovea [6–9], this semantic preview effect is stably reported in studies using Chinese language [10–14].

Recently, researchers used event-related brain potentials (ERP) techniques to study the parafoveal process in word-pair reading and sentence reading. In most word-pair studies, one word was present in participants’ foveal vision and the other word was present in the parafoveal vision. Participants were asked to read the foveal word first and then make saccade to the parafoveal word. Participants’ eye movements and EEG were simultaneously recorded during reading, and their eye-fixation-related potentials (EFRP) were computed to investigate the semantic processing of the parafoveal word [15–17]. Using this method, Baccino and Manunta [15] manipulated the type of word located in the right parafovea to yield three conditions: semantically related (horse-mare), semantically unrelated (horse-table) and non-word (horse-twsui). The EFRP results revealed a more positive P2 time locked to the onset of fixation on the foveal prime word (horse) in the semantically related preview condition than in the unrelated preview condition, which showed a semantic processing of the parafoveal word. However, several other studies did not observe the parafoveal semantic effects in word-pair reading [16,17]. For example, In Dimigen’s study [17], participants were asked to read 5 German words fluently from left to right, and the results did not reveal the parafoveal semantic effect.

In studies using ERP to investigate the parafoveal processing during sentence reading, the RSVP-flanker paradigm was mainly used [18–20]. In this paradigm, sentences were presented word by word at the center of the screen. Each word (n) was always flanked bilaterally 2° by the next word (n+1) on its right and by the preceding word (n-1) on its left. Thus, three words (triad) were presented in each display. To ensure the flanker words were in the readers’ parafoveal vision field, readers were asked to maintain their fixation on the word in the center of the screen and avoid eye movements. By manipulating the semantic congruency of the word in the parafovea and analyzing the related ERP components that time-locked to the onset of that parafoveal word, researchers inferred whether the parafoveal semantic information could be extracted and integrated. For example, using German sentences, Barber et al. [19] reported that the incongruent words evoked a more negative response of the N400 component compared with congruent ones time-locked to critical triads, in which the critical word was presented as right flanker. This study indicates that the parafoveal word’s semantic information can be integrated during sentence reading. Furthermore, Barber et al. [20] investigated how the cognitive load affects parafoveal semantic processing in sentence reading by manipulating the Stimulus Onset Asynchrony (SOA) of word presentation and contextual constraint on critical words. The results showed a parafoveal N400 congruity effect for both high-constraint sentences (≥ 0.7) and low-constraint sentences (≤ 0.4), when presentation rate was slow. However, when presentation rate was fast, the parafoveal N400 congruity effect was only found for high-constraint sentences, not for low-constraint sentences. Because cognitive resources are more limited under short SOA and/or low contextual constraint, this result suggests that the semantic processing of parafoveal words is affected by current cognitive resources.

The parafoveal N400 effect in the sentence studies clearly showed that the semantic information of the parafoveal word can be extracted and integrated during sentence reading. However, as most studies did not control the congruency between the foveal and parafoveal words that were presented on the critical triad, it is unclear whether the semantic integration happened at the sentence level or just at the word-pair level. For example, in one illustration sentence in Barber’s study [20], “Mary bought her new bike/head last week because it was on sale”, the parafoveal target “bike/head” is congruent/incongruent with the foveal pre-target “new”, both of which were presented in the critical triad. Thus, to detect such incongruence, participants did not necessarily integrate the parafoveal information with the global semantic context of the sentence; rather, all they needed to do was to process a word-pair composed of the foveal and parafoveal words. Thus, more rigorous study is needed to test whether parafoveal integration can be conducted at the sentence level.

Compared with the integration of information between word-pair, integration of the target word with global context (the semantic context of the sentence or discourse level before the pre-target word) may require more cognitive resources. Whether such integration could occur immediately is a critical and long-standing issue in studies of language comprehension. Based on the previous literature, most studies suggested that the semantic integration with global context can be conducted immediately. For instance, Zhou et al. [21] investigated semantic integration at different levels of syntactic hierarchy during sentence reading. The results showed that integrating the object noun with the classifier (the word prior to the object noun) or with the verb from previous context occurred in the same N400 time window. This suggests that integration with global semantic context can be conducted as rapidly as integration at the word-pair level during sentence reading. Furthermore, previous studies also demonstrated that semantic integration with background information can start immediately during discourse reading [22–24]. However, given the concern that the processing of a word starts when it is in parafoveal vision, to study the integration of parafoveal semantic information with global context is important for us to have a full understanding of the time course of the integration process.

The research on the integration of semantic information in parafovea can also help us to distinguish between two types of cognitive control model of eye movement in text reading: serial attention shift (SAS) models and guidance by attentional gradient (GAG) models. The SAS models such as the E-Z Reader model assumes that lexical semantic processing is guided by the shifting of attentional resources in a serial mode, and the semantic extraction of word n+1 occurs after the successful semantic access of word n [25–27]. However, the GAG models such as saccade-generation with inhibition by foveal targets (SWIFT) model suggests that the attentional resources reach the maximum around the fixation and then decrease gradually to both sides, and semantic processing is distributed over a number of words in parallel [28,29]. Therefore, the critical difference between these two types of model is whether the semantic information of parafoveal and foveal words can be processed in parallel. Consequently, investigating whether the semantic integration of parafoveal words and foveal words could occur simultaneously will shed light on the eye movement control models.

In the present study, the RSVP-flanker paradigm was employed to examine semantic integration of parafoveal words during Chinese sentence reading. To ensure that the parafoveal N400 effect of congruity can only be triggered by integration of the critical noun in the parafovea at the sentence level, verbs in the sentence were manipulated to yield either a congruent or incongruent context for the critical noun, which was presented at a distance of 4 or 5 characters after the verbs. Thus, the characters (including the critical noun) in the critical triad were the same across the two conditions. Meanwhile, the critical noun in parafoveal vision and the middle character in the critical triad were always congruent. Furthermore, we were interested in whether this parafoveal integration could be conducted within only 100 ms of perception. Thus, we used a mask to substitute for the parafoveal target after it was presented for 100 ms, so that we could precisely control the duration of the parafoveal perception of the target word. Finally, the contextual constraint ratings of the middle character and right parafoveal character in the critical triad and the cloze predictability rating of the middle character in the critical triad were calculated to rule out the possibility that the parafoveal N400 effect was elicited by the difference in contextual constraints or the processing of middle characters in critical triads. If a more negative N400 time-locked to the onset of the critical triads could be elicited in the incongruent condition compared with the congruent condition, it would suggest that semantic information in parafovea could be integrated at the sentence level within a short perception time.

## Materials and Method

### Ethics Statement

This study was approved by the Psychology Research Ethics Committee of South China Normal University. The participants provided written informed consent prior to the experiment.

### Participants

Twenty right-handed students (5 males; mean age = 22.7 years) from South China Normal University were paid 30 yuan to participate in the ERP experiment. All were native speakers of Mandarin Chinese, had no reading disabilities, and had normal or corrected-to-normal vision.

### Materials

A total of 84 sets of Chinese sentences were constructed for this study (see Table 1 for examples). These sentences had a similar syntactic structure (subject + verb + adjective/quantifier + object +…). In each set of sentences, we manipulated the verbs to yield either semantically congruent or incongruent context for the critical single-character noun (object). In particular, the interval between these critical nouns and verbs was controlled to be 4 or 5 characters. In addition, the critical noun was always followed by an additional 4 to 8 characters.

**Table 1:** Example sentences with English translations.

Condition	Sample sentences
	(1)韩梅 (2)① 摘掉了 (3)那 (4)② 几 只 瓜 * (5)并 (6)装进 (7)袋子。
Congruent	(1)Han Mei (2)① picked (3)there (4)② the melons * (5)and (6)put into (7)a bag.
	*Han Mei picked the melons there and put them into a bag*.
	(1)韩梅 (2)① 捕获了 (3)那 (4)② 几 只 瓜 * (5)并 (6)装进 (7)袋子。
Incongruent	(1)Han Mei (2)① captured (3)there (4)② the melons * (5)and (6)put into (7)a bag.
	*Han Mei captured the melons there and put them into a bag*.

*, The target noun

①, The verb that is congruent or incongruent with the target noun

②, The critical triad containing the critical noun as a right flanker.

The numbers in parentheses mean the order of the corresponding segments between experimental sentences and their English translations.

Strokes and word frequency of verbs were matched to avoid possible influences on ERP results after the critical triads. The average strokes of verbs was 17.87 (*SD* = 4.22) for the congruent and 17.65 (*SD* = 3.77) for the incongruent condition. The average frequency of verbs was 6.42 (*SD* = 8.73) per million for the congruent and 6.37 (*SD* = 12.21) per million for the incongruent condition. Paired samples t-tests showed no significant difference in strokes, *t*(83) < 1, or word frequency, *t*(83) < 1.

Two different sentence lists were counterbalanced across participants so that each participant read 42 sentences per condition and no sentence frame was presented twice. In each list, the 84 sentences were divided in four blocks and presented randomly. Each participant conducted 10 practice sentences and was assigned to one list.

### Rating

Two semantic plausibility rating studies were conducted to determine the validity of our sentences. Twenty-four participants who did not take part in the ERP experiment were recruited to rate the semantic plausibility of the full sentences on a 5-point scale (ranging from 1 = extremely unacceptable to 5 = fully acceptable). The average ratings for the congruent and incongruent complete sentences were 4.16 (*SD* = 0.37) and 1.66 (*SD* = 0.40), respectively. Another 24 participants rated the first part of the sentences, up to and including the critical noun. The average scores were 4.18 (*SD* = 0.32) for the congruent and 1.65 (*SD* = 0.34) for the incongruent condition. The results revealed significant differences between the congruent and incongruent conditions, both for the complete sentences, *t*(83) = 40.09, *p*< .001, and for the first part of sentences, *t*(83) = 48.46, *p*< .001.

In addition, as mentioned in the introduction, three rating studies were conducted to determine (1) the contextual constraint of the critical noun location, (2) the contextual constraint of the location before the critical noun (i.e., the location of the middle character in critical triads), (3) the cloze predictability for the character before the critical noun (i.e., the middle character in critical triads). Forty-four participants were recruited to rate the contextual constraint of the critical noun location. They were given the first part of the experimental sentences up to (not including) the critical nouns and were asked to provide the next character in the sentence. We calculated the percentage of times that each noun appeared in each sentence, and took the highest one as the indicator of contextual constraint. The average contextual constraint for the congruent and incongruent sentences was 0.47 (*SD* = 0.21) and 0.51 (*SD* = 0.22), respectively; no significant difference was found across the two conditions, *t*(83) = 1.31, *p*> .1. Thirty-six participants were asked to rate the contextual constraint of the location before the critical noun and the cloze predictability for the character in this location. The results revealed that average contextual constraint was 0.41 (*SD* = 1.17) for the congruent and 0.41 (*SD* = 1.18) for the incongruent condition, and no significant difference was found, *t*(83) < 1. Similarly, the average cloze predictability was 0.27 (*SD* = 0.25) for the congruent and 0.27 (*SD* = 0.26) for the incongruent condition, with no significant difference, *t*(83)< 1.

### Procedure

**Fig 1:**
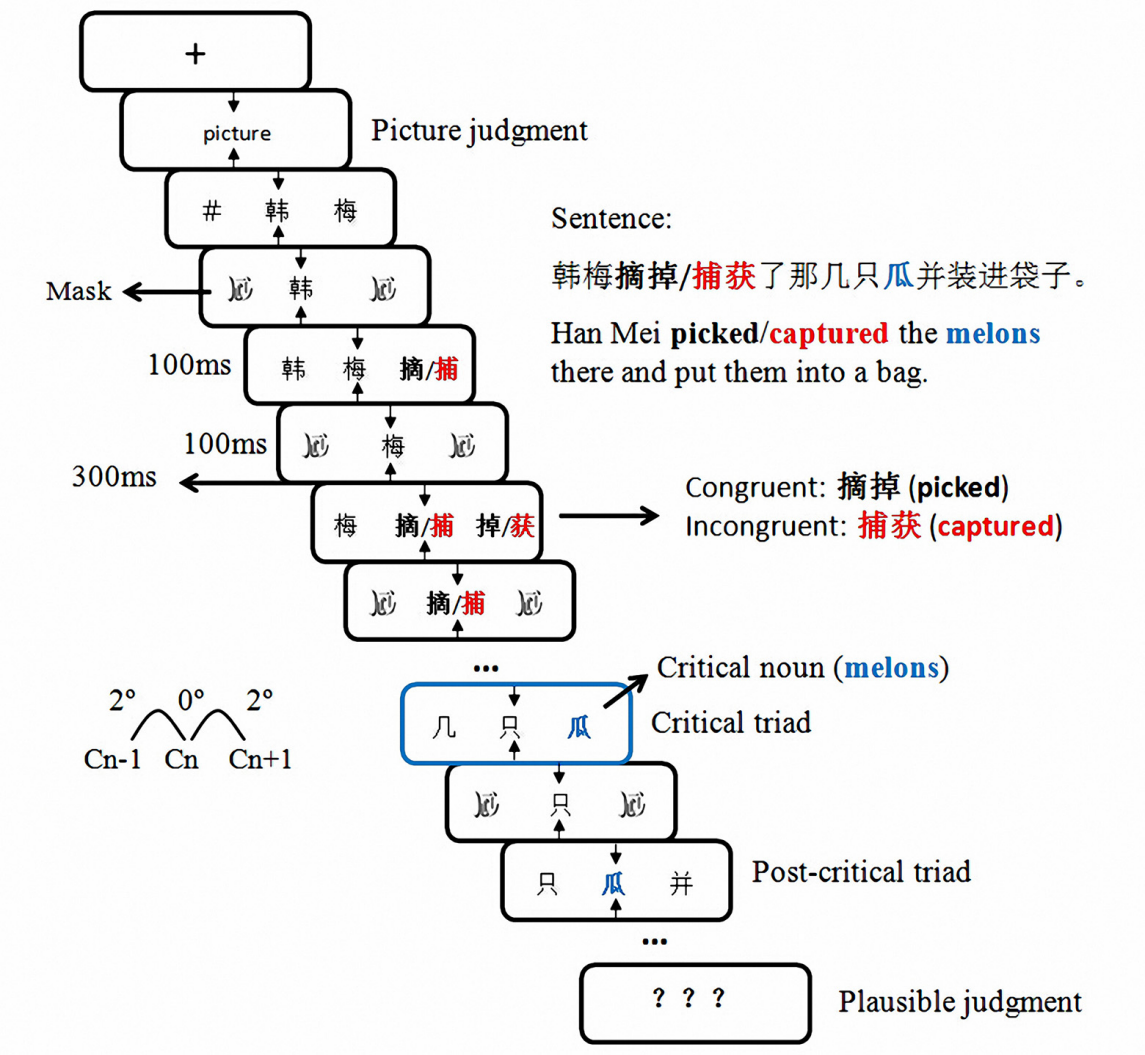
Sentence presentation procedure. Sentences were presented character by character in the center of the screen, flanked by the previous character to the left and the subsequent character to the right. The context verb was either congruent (*picked*) or incongruent (*captured*) with the critical noun (melons) in the sentence. Participants were asked to keep their gaze fixated on the central character and to judge the plausibility of the sentence.

A 17-inch CRT monitor was used to display the stimuli, and the monitor was set to a refresh rate of 75 Hz. The IBM Think Centre (9215) computer and E-Prime software package (Psychology Software Tools, Pittsburgh, PA) were used for stimulus presentation and response collection. Prior to the experiment, participants were given the experimental instructions. As shown in Fig 1, in order to keep a central fixation location, a picture subtended about 0.5° of visual angle was presented in the center of the screen for 1000 ms before the presentation of each sentence. Participants were asked to distinguish them from each other and press the corresponding button for each picture. It is worth noting that with enough practice, participants pressed the button without looking at the keyboard. Sentences were presented character by character at the center of the screen in 13 to 18 triads. Each character (n) was flanked bilaterally by the next character of the sentence (n+1) to the right, and the previous character (n-1) to the left. Characters in the center of the screen were presented for 200ms, followed by a 300 ms blank interval; characters in the parafoveal region were presented for 100 ms and then were replaced by a mask which also was presented for 100ms.

All characters were presented in black against a gray background. The typeface of the characters was “Kai”. Each character was subtended about 1.35° of visual angle at the viewing distance of 85 cm. Characters were presented with one empty character space between them. Thus, the left edge of the right parafoveal character was presented about 2° (1.5 character spaces×1.35°) away from the screen center. To keep the participants’ fixations on the center character, two arrows up and below the location of the center character were added in each triad.

After each sentence, participants were asked to decide whether the sentence was plausible or not by pressing the “F” or “J” button on a keyboard. The buttons were counterbalanced among participants. There was a pause between every two sentences and the participants could press the “space” button to continue after they had enough rest. It should be noted that although participants were not explicitly asked to ignore flankers, they were told to maintain focus on the center character and avoid eye movements and blinks during the presentation of sentences. The whole experiment for each participant lasted around 40 min.

### EEG Recording and Data Analysis

Participants were tested individually in a sound-attenuating, electrically shielded booth after being fit with an electrode cap (Acticap, Brain Products). EEG were recorded with 40 tin electrodes (10–20 System) and four electro-oculogram (EOG) electrodes. Signals were referenced online to left mastoid and re-referenced offline to the average of the two mastoid electrodes. The AFz electrode on the cap served as ground. Impedance was kept below 5 kΩ for all electrodes. The EEG and EOG signals were sampled at 500 Hz and filtered digitally with a .05 to 30 Hz band pass offline. Epochs of interest were selected time-locked to the onset of the critical triads with a 200 ms pre-onset baseline window and a 1200 ms window after their onset. Ocular and movement artifacts (> ±80μV) were excluded from further analyses.

Following Barber et al. [20], the baseline interval before the critical triad was also used to analyze the foveal N400 effect evoked by the post-critical triad (the triad following the critical triad), in which the critical noun was presented at fixation. This ensures that the earlier parafoveal N400 effects do not influence the baseline correction for the analysis of the following foveal N400 effect. As the classical N400 time window was at 300~500 ms after the onset of the critical word and the time interval between the onset of the post-critical triad and the onset of the critical triad was 500 ms, the time window of the foveal N400 effect that was elicited by the post-critical triads should be at 800~1000 ms after the onset of the critical triad. Therefore, the present study extracted the average amplitudes of the waveform at both 300~500 ms and 800~1000 ms after the critical triad for the ANOVA analysis. It is worth noting that the parafoveal N400 effect is unrelated to the post-critical triads because the time window of the parafoveal N400 effect was at 300~500 ms, during which time the post-critical triad had not been presented (SOA = 500 ms).

**Fig 2:**
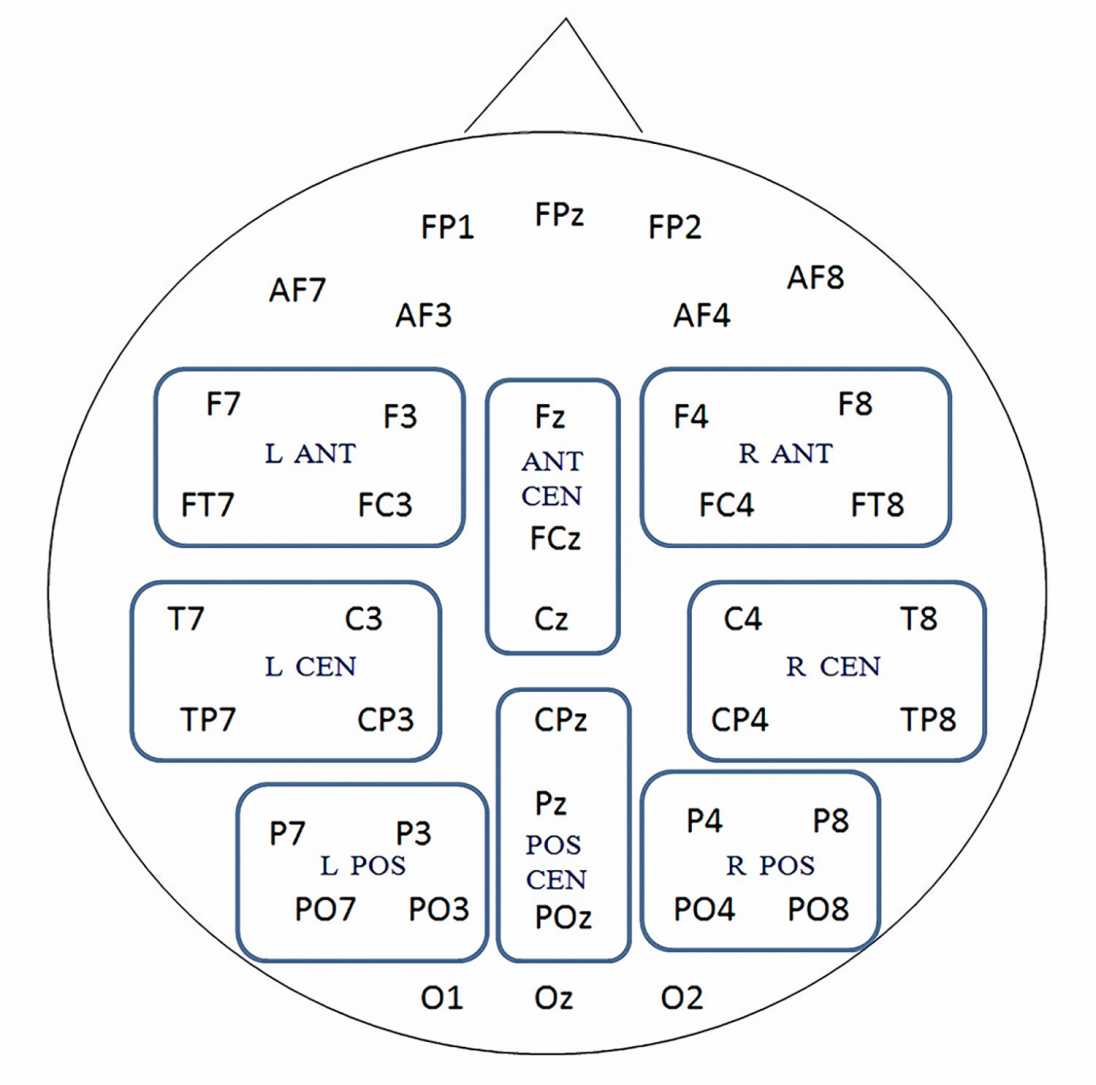
Forty recorded electrodes and 8 AOIs for ANOVA. ANT, anterior; CEN, central; POS, posterior; L, left hemisphere; R, right hemisphere.

For each time window, repeated-measures ANOVAs with Greenhouse-Geisser correction were carried out for eight electrode regions. The ANOVA was performed with two factors: condition (congruent and incongruent) and area of interest (AOI, of which there were eight). Each AOI comprised 4 or 3 electrodes (see Fig 2): left anterior (F7, F3, FT7, FC3), right anterior (F4, F8, FC4, FT8), left central (T7, C3, TP7, CP3), right central (C4, T8, CP4, TP8), left posterior (P7, P3, PO7, PO3), right posterior (P4, P8, PO4, PO8), anterior central (Fz, FCz, Cz) and posterior central (CPz, Pz, POz). Data were averaged within each AOI before statistical analysis.

## Results

### Behavior Results

The mean accuracy rate of picture judgment was 97.68% (*SD* = 3.59%), suggesting that participants’ performance was almost perfect in fixating the central location before the presentation of each sentence. All participants scored 80% or better in response to the semantic plausibility judgment task, averaging a 90.48% (SD = 6.95%) hit rate for the congruent condition and 94.88% (SD = 4.78%) correct rejection rate for the incongruent condition. The results indicated that participants understood most of the sentences correctly. The average reaction time (RT) was 635.46 ms (SD = 186.73) in the congruent condition and 543.26 ms (SD = 159.50) in the incongruent condition. Paired samples t-tests revealed that the correct rejection rate for incongruent sentences was significantly higher, *t*(19) = 2.12, *p*< .05, and the RT of correct rejections to incongruent sentences was significantly faster, *t*(19) = 4.41, *p*< .001, than the hit rate and RT for congruent sentences. The results may suggest that the detection of semantic incongruence is easier than confirmation of semantic congruence.

### ERP Results

**Fig 3:**
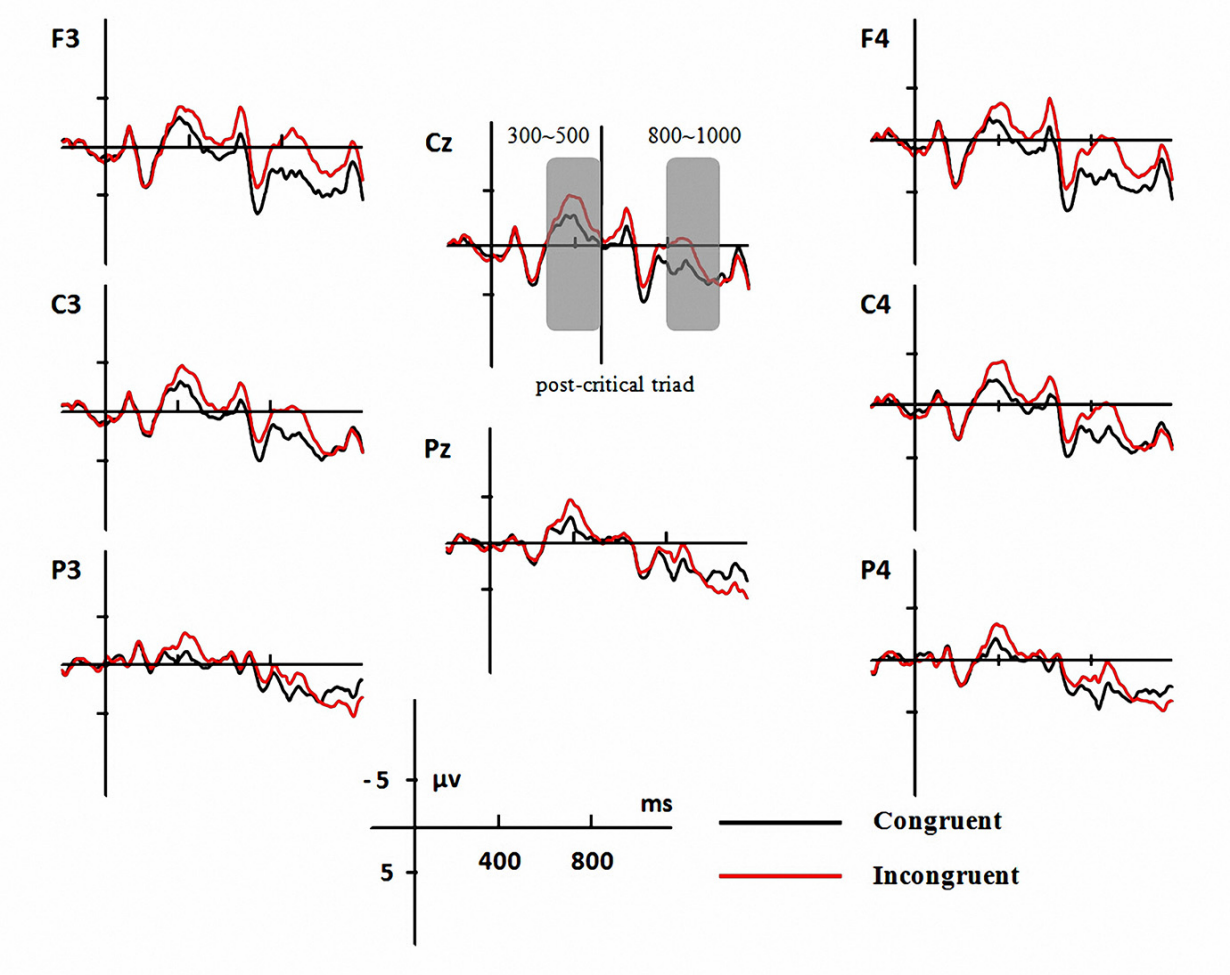
Grand average of ERP. Average EEG waveforms from the onset of the critical triads up to 1200 ms thereafter for congruent condition (black line) and incongruent condition (red line) at 8 representative electrodes, one for each AOI. Negativity is plotted upwards.

The grand-average ERP of nine representative electrodes time-locked to the critical triads are presented in Fig 3. There was two clear negative deflections at 300~500 ms and 800~1000ms. With comprehensive consideration of their polarity and latency, they can be confirmed as N400 component evoked by the critical triads and N400 component evoked by the post-critical triads respectively. The mean amplitude of each condition in each AOI was presented in Table 2.

**Table 2:** Mean amplitude across conditions in each AOI in the analyzed time window.

AOI		300~500ms		800~1000ms	
		Congruent	Incongruent	Congruent	Incongruent
	ANT	-1.41(0.30)	-2.39(0.36)	2.25(0.51)	-0.33(0.45)
L	CEN	-1.01(0.22)	-1.99(0.30)	1.89(0.48)	0.55(0.53)
	POS	-0.33(0.28)	-1.29(0.37)	1.19(0.50)	0.42(0.65)
	ANT	-0.87(0.25)	-2.18(0.43)	3.42(0.43)	0.28(0.44)
R	CEN	-0.96(0.22)	-2.24(0.39)	2.95(0.49)	0.61(0.54)
	POS	-0.52(0.23)	-1.68(0.37)	2.44(0.53)	0.81(0.70)
ANT CEN		-1.75(0.39)	-3.48(0.44)	2.89(0.58)	-0.05(0.63)
POS CEN		-1.15(0.34)	-2.56(0.47)	2.55(0.65)	1.31(0.87)

ANT, anterior; CEN, central; POS, posterior; L, left hemisphere; R, right hemisphere. Standard error mean is shown in brackets.

#### Parafoveal N400 effect (300~500ms)

**Fig 4:**
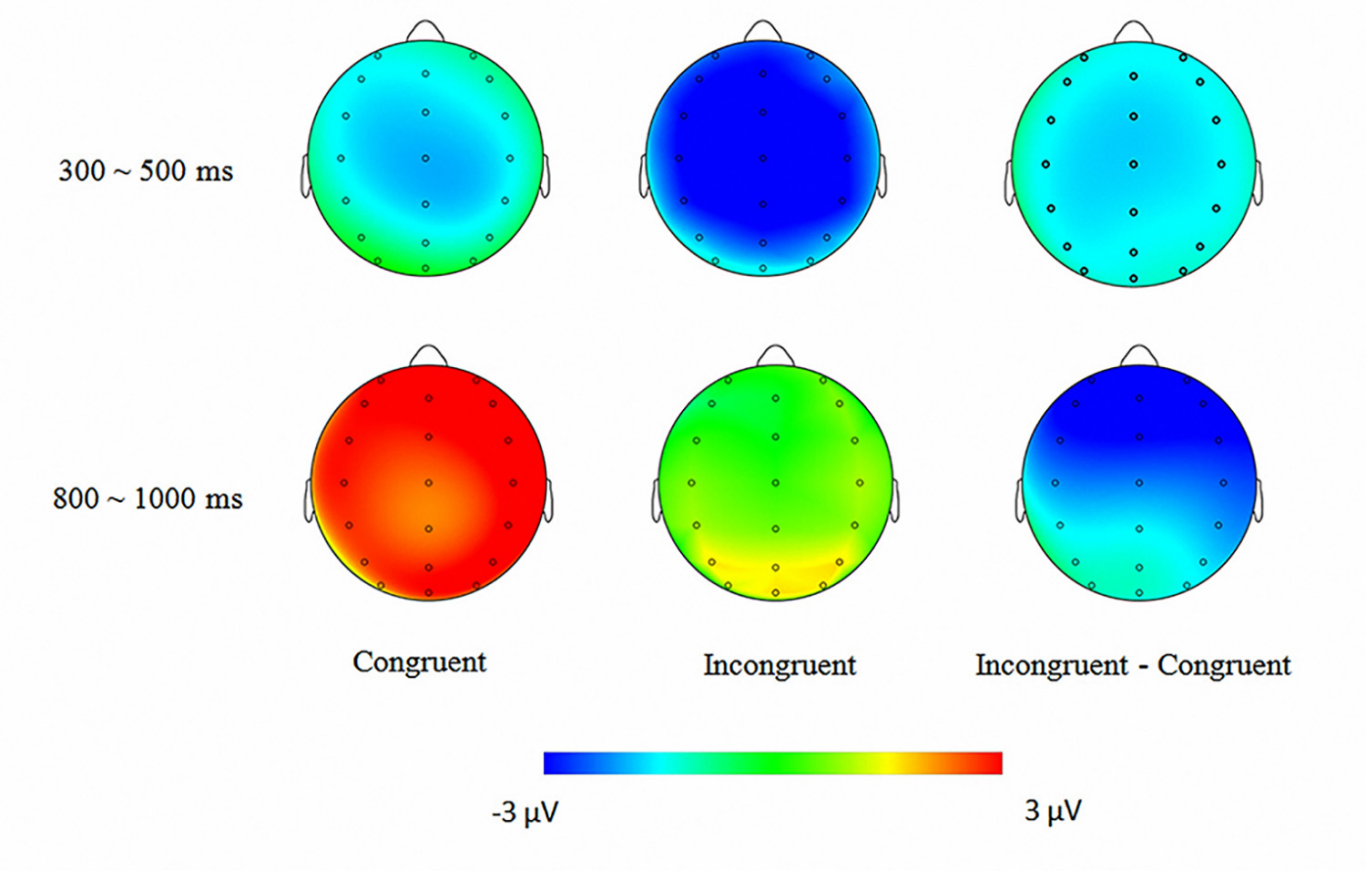
Scalp distributions in the analyzed time window. 300~500 ms was the N400 time window evoked by the critical triads and 800~1000 ms was the N400 time window evoked by the post-critical triads.

The ANOVA of amplitudes showed a significant main effect of condition, *F*(1, 19) = 22.25, *p*< .001, *η* ^*2*^ = 0.54, with no other main effect or interaction. Separate ANOVAs at each AOI showed that the condition effect was significant at all eight AOI (see Fig 4), all *Fs*> 9.89, *ps*< .01, with the incongruent sentences eliciting more negative N400 compared with congruent ones.

#### Foveal N400 (800~1000ms)

The ANOVA of amplitudes showed a significant main effect of condition, *F*(1, 19) = 17.52, *p*< .005, *η* ^*2*^ = 0.48, and a significant condition×AOI interaction, *F*(7, 133) = 12.69, *p*< .001, *η* ^*2*^ = 0.40. Separate ANOVAs at each AOI showed that the semantic condition effect was significant at six AOI, namely left anterior, left central, right anterior, right central, right posterior, and anterior central, all *Fs*> 6.73, *ps*< .05. The left posterior and posterior central AOI showed a marginally significant effect, all *Fs*> 3.12, *ps*< .1. As in the 300~500 ms time window, the N400 was more negative for incongruent sentences.

## Discussion

The present study explored the semantic integration of parafoveal words in Chinese sentence reading by using ERP technique with the RSVP-flanker paradigm. The results showed that the N400 component time-locked to the onset of critical triads was more negative in the semantically incongruent condition than in the semantically congruent condition. Because the parafoveal word in each screen was quickly replaced by a mask after it was presented for 100 ms, the results of our study indicate that the semantic information from parafovea can be extracted within only 100 ms of perception and integrated at the sentence level.

As is well known, contextual integration of word meaning can occur at different levels, namely word-pair, sentence and discourse, and these different levels of integration may relate to different mechanisms [30]. Studies on different levels of integration in parafovea could provide a more rigorous test of the immediacy of semantic integration. Previous studies that used sentences as materials showed that an incongruent target word could be detected when it was in the parafoveal region, but such integration processing may actually happen on the word-pair level rather than on the sentence level. Such a possibility was ruled out in the current study by strictly controlling the distance between the context verb and the critical noun. Specifically, the context verb that was congruent or incongruent with the critical noun was presented several triads before the critical triad. Thus, to detect whether the critical noun in the parafovea was congruent or incongruent in the sentence, participants had to integrate it with global semantic context maintained in their working memory. The parafoveal N400 effect we found in the current study, along with those observed in previous studies, thus provides strong evidence that parafoveal semantic information can be integrated at both the word-pair and sentence levels.

The time course of parafoveal semantic processing is important to distinguish the models of how attention shifts during text reading [31,32]. As mentioned in the introduction, the E-Z reader model assumes that semantic processing of words is guided by the shifting of attentional resources in a serial mode. However, the SWIFT model suggests that semantic processing is distributed over a number of words via a spatially distributed of attention. The present study demonstrated that the parafoveal N400 effect occurred within 300~500 ms and with the centro-parietal distribution, which is similar to the classical N400 effect of semantic integration in studies in which words were presented at fixation. Thus, the parafoveal N400 is essentially similar to the classical N400 effect, which implies that the parafoveal semantic integration could be conducted as early as foveal semantic integration, and therefore the foveal and parafoveal words may be processed in parallel. This supports the SWIFT model arguing for distributed word recognition with gradient allocation policies.

In the present study, we observed a foveal N400 congruency effect time-locked to the post-critical triads in which the critical nouns were in the fovea. The results is consistent with previous study [20], and suggesting the spill over effect of the parafoveal N400 effect. However, compared with the N400 congruency effect found in studies using the traditional RSVP paradigm, the foveal N400 effect we observed was mainly distributed in frontal regions (i.e., anterior N400 effect). Relatively speaking, the distribution of the parafoveal N400 effect was widespread and posterior, which was more consistent with the one found in studies using the RSVP paradigm. This difference is very interesting. One possible explanation is that unlike the semantic integration of directly fixated words, the semantic integration of parafoveal words may contain two different stages in a long-lasting process. The first stage mainly occurs at a lexical semantic level, whereas the second stage biases toward situation establishment, which involves more world knowledge. Previous studies indicated that the scalp distribution of N400 effects that reflected situation establishment was more anterior than the distribution that reflected semantic processing [33]. Similarly, the foveal N400 effect of the present study was more anterior, which may indicate participants put more weight on the process of situation establishment. In contrast, the parafoveal N400 effect was more widespread, which may reflect the early integration process at a semantic level. Another possible explanation is that the anterior N400 is partly linked to familiarity-driven cognition of critical nouns in post-critical triads. Previous studies demonstrated that the anterior 400 effect reflected the familiarity of words: new words would elicit more negative anterior 400 compared with old ones [34,35]. Thus, the anterior N400 of post-critical triads may be linked to the critical nouns in the congruent condition being more familiar, because the congruent context facilitated the identification of these characters. More studies should be conducted to investigate the above-mentioned possibilities in the future.

Previous studies indicated that a semantic parafoveal-on-foveal effect was more consistently found in studies using the RSVP-flanker paradigm than studies using the boundary paradigm [19,20,36–38]. One possible explanation of the inconsistency is that in the boundary paradigm, parafoveal perception could be interrupted when readers’ eyes moved to the target location. However, in the RSVP-flanker paradigm, words are always presented for a fixed time duration, and followed by a long interval before the subsequent word appears. Therefore, readers are able to continue their perception of the parafoveal word through the afterimage of that word. In the present study, the perception of parafoveal words was interrupted, because the parafoveal words were immediately replaced by a mask after presentation; however, a parafoveal N400 effect was still observed. This indicates that the perception interruption of parafoveal words is not a main factor in the inconsistent results between the boundary paradigm and the RSVP-flanker paradigm. Rather, this inconsistency may be due to participants having more cognitive resources for parafoveal processing in the RSVP-flanker paradigm [20].

It should be noted that the parafoveal N400 effect we observed was unlikely to be elicited by lower lexical processing of the parafoveal characters in critical triads. This is because the characters in critical triads were the same across the two conditions. Moreover, the parafoveal N400 cannot be accounted for by the contextual constrain or the processing of middle characters in critical triads, because the results of these ratings showed no significant difference across the two conditions. With all these controls, we have more confidence to say that the parafoveal N400 effect observed was elicited by the semantic integration of the parafoveal word at the sentence level.

In RSVP-flanker paradigm, it is important to ensure that participants were able to fixate on central characters during sentence presentation. Thus, we carried out an eye movement experiment with the identical design and materials to confirm the flanker character was indeed presented at parafoveal vision. An SR Research Eyelink 1000 system was used to track participants’ eye movements with a sample rate of 1000 Hz. Only movements of the right eye were recorded, although viewing was binocular. Ten university students participated in the eye movement experiment. Because we were only concerned about locations of participants’ fixation while the sentence was presented, three regions of interest (ROI) were set with each character. The side of each ROI was set as 1.5 times the character length. We extracted the locations of participants’ fixations during sentence presentation and calculated the percentage of the fixations in each ROI. The results showed that the percentages of fixations located on the ROI of the left, middle and right character were 0.09%, 93.69% and 0.6% respectively. This suggests that participants were able to fixate on the central character in the present paradigm.

In conclusion, the present study provides direct evidence that the semantic information from parafovea can be integrated at the sentence level during Chinese reading. Moreover, the results indicate that the semantic integration of the parafoveal word can be conducted within only 100 ms of perception. The important theoretical implication of this study is that global context factors need to be considered in future studies’ constructions of eye movement control models on parafoveal processing.

Note: M37, the congruent condition; M47, the incongruent condition.

## Supporting Information

S1 Appendix Eighty-four sets of experimental sentences. (DOCX)

S2 Appendix Mean amplitude of 300~500 ms of all participants. Note: M37, the congruent condition; M47, the incongruent condition.
(SAV)

S3 Appendix Mean amplitude of 800~1000 ms of all participants. (SAV)

## References

[1] Paterson KB, McGowan VA, White SJ, Malik S, Abedipour L, Jordan TR. Reading direction and the central perceptual span in Urdu and English. PloS one. 2014; 9: e88358 10.1371/journal.pone.0088358 24586316 PMC3934859

[2] Chace KH, Rayner K, Well AD. Eye movements and phonological parafoveal preview: effects of reading skill. Canadian Journal of Experimental Psychology. 2005; 59: 209–217. 16248500 10.1037/h0087476

[3] Juhasz BJ, White SJ, Liversedge SP, Rayner K. Eye movements and the use of parafoveal word length information in reading. Journal of Experimental Psychology: Human Perception and Performance. 2008; 34: 1560–1579. 10.1037/a0012319 19045993 PMC2668122

[4] Miellet S, Sparrow L. Phonological codes are assembled before word fixation: Evidence from boundary paradigm in sentence reading. Brain and Language. 2004; 90: 299–310. 15172547 10.1016/S0093-934X(03)00442-5

[5] Starr M, Inhoff A. Attention allocation to the right and left of a fixated word: Use of orthographic information from multiple words during reading. European Journal of Cognitive Psychology. 2004; 16: 203–225.

[6] Rayner K, Balota DA, Pollatsek A. Against parafoveal semantic preprocessing during eye fixations in reading. Canadian Journal of Psychology. 1986; 40: 473–483. 3502884 10.1037/h0080111

[7] Altarriba J, Kambe G, Pollatsek A, Rayner K. Semantic codes are not used in integrating information across eye fixations in reading: Evidence from fluent Spanish-English bilinguals. Perception & Psychophysics. 2001; 63: 875–890. 11521853 10.3758/bf03194444

[8] Rayner K, Morris RK. Eye movement control in reading: Evidence against semantic preprocessing. Journal of Experimental Psychology: Human Perception and Performance. 1992; 18: 163–172. 1532186

[9] Hyönä J, Häikiö T. Is emotional content obtained from parafoveal words during reading? An eye movement analysis. Scandinavian Journal of Psychology. 2005; 46: 475–483. 16277648 10.1111/j.1467-9450.2005.00479.x

[10] Yan M, Richter EM, Shu H, Kliegl R. Readers of Chinese extract semantic information from parafoveal words. Psychonomic Bulletin & Review. 2009; 16: 561–566. 19451385 10.3758/PBR.16.3.561

[11] Yan M, Zhou W, Shu H, Kliegl R. Lexical and sublexical semantic preview benefits in Chinese reading. Journal of Experimental Psychology: Learning, Memory, and Cognition. 2012; 38: 1069–1075. 10.1037/a0026935 22369254

[12] Yang J, Wang S, Tong X, Rayner K. Semantic and plausibility effects on preview benefit during eye fixations in Chinese reading. Reading and Writing. 2012; 25: 1031–1052. 22593624 10.1007/s11145-010-9281-8 PMC3337412

[13] Yang J, Li N, Wang S, Slattery TJ, Rayner K. Encoding the target or the plausible preview word? The nature of the plausibility preview benefit in reading Chinese. Visual Cognition. 2014; 22: 193–213. 24910514 10.1080/13506285.2014.890689 PMC4043386

[14] Zhou W, Kliegl R, Yan M. A validation of parafoveal semantic information extraction in reading Chinese. Journal of Research in Reading. 2013; 36: 51–63.

[15] Baccino T, Manunta Y. Eye-fixation-related potentials: Insight into parafoveal processing. Journal of Psychophysiology. 2005; 19: 204–215.

[16] Simola J, Holmqvist K, Lindgren M. Right visual field advantage in parafoveal processing: Evidence from eye-fixation-related potentials. Brain and language. 2009; 111: 101–113. 10.1016/j.bandl.2009.08.004 19782390

[17] Dimigen O, Kliegl R, Sommer W. Trans-saccadic parafoveal preview benefits in fluent reading: A study with fixation-related brain potentials. NeuroImage. 2012; 62: 381–393. 10.1016/j.neuroimage.2012.04.006 22521255

[18] Barber HA, Ben-Zvi S, Bentin S, Kutas M. Parafoveal perception during sentence reading? An ERP paradigm using rapid serial visual presentation (RSVP) with flankers. Psychophysiology. 2010; 48: 523–531. 10.1111/j.1469-8986.2010.01082.x 21361965 PMC4075191

[19] Barber HA, Doñamayor N, Kutas M, Münte T. Parafoveal N400 effect during sentence reading. Neuroscience Letters. 2010; 479: 152–156. 10.1016/j.neulet.2010.05.053 20580772 PMC4096702

[20] Barber HA, Meij M, Kutas M. An electrophysiological analysis of contextual and temporal constraints on parafoveal word processing. Psychophysiology. 2013; 50: 48–59. 10.1111/j.1469-8986.2012.01489.x 23153323 PMC4096715

[21] Zhou X, Jiang X, Ye Z, Zhang Y, Lou K, Zhan W. Semantic integration processes at different levels of syntactic hierarchy during sentence comprehension: An ERP study. Neuropsychologia. 2010; 48: 1551–1562. 10.1016/j.neuropsychologia.2010.02.001 20138898

[22] Berkum JJ, Hagoort P, Brown CM. Semantic integration in sentences and discourse: Evidence from the N400. Journal of Cognitive Neuroscience. 1999; 11: 657–671. 10601747 10.1162/089892999563724

[23] Wang S, Chen HC, Yang J, Mo L. Immediacy of integration in discourse comprehension: Evidence from Chinese readers’ eye movements. Language and Cognitive Processes. 2008; 23: 241–257.

[24] Van Berkum JJ, Zwitserlood P, Hagoort P, Brown CM. When and how do listeners relate a sentence to the wider discourse? Evidence from the N400 effect. Cognitive brain research. 2003; 17: 701–718. 14561457 10.1016/s0926-6410(03)00196-4

[25] Rayner K, Li X, Pollatsek A. Extending the E-Z reader model of eye movement control to Chinese readers. Cognitive Science. 2007; 31: 1021–1033. 10.1080/03640210701703824 21635327

[26] Reichle ED, Pollatsek A, Rayner K. E-Z Reader: A cognitive-control, serial-attention model of eye-movement behavior during reading. Cognitive Systems Research. 2006; 7: 4–22.

[27] Reichle ED, Warren T, McConnell K. Using E-Z Reader to model the effects of higher level language processing on eye movements during reading. Psychonomic Bulletin & Review.2009; 16: 1–21. 19145006 10.3758/PBR.16.1.1 PMC2629133

[28] Engbert R, Longtin A, Kliegl R. A dynamical model of saccade generation in reading based on spatially distributed lexical processing. Vision Research. 2002; 42: 621–636. 11853779 10.1016/s0042-6989(01)00301-7

[29] Engbert R, Nuthmann A, Richter EM, Kliegl R. SWIFT: A dynamical model of saccade generation during reading. Psychological Review. 2005; 112: 777–813. 16262468 10.1037/0033-295X.112.4.777

[30] Bemis DK, Pylkkänen L. Simple composition: A magnetoencephalography investigation into the comprehension of minimal linguistic phrases. The Journal of Neuroscience. 2011; 31: 2801–2814. 10.1523/JNEUROSCI.5003-10.2011 21414902 PMC6623787

[31] Rayner K, Liversedge SP, White SJ. Eye movements when reading disappearing text: The importance of the word to the right of fixation. Vision research. 2006; 46: 310–323. 16085229 10.1016/j.visres.2005.06.018

[32] Hohenstein S, Laubrock J, Kliegl R. Semantic preview benefit in eye movements during reading: A parafoveal fast-priming study. Journal of Experimental Psychology: Learning, Memory, and Cognition. 2010; 36: 1150–1170. 10.1037/a0020233 20804291

[33] Chwilla DJ, Kolk HH. Accessing world knowledge: evidence from N400 and reaction time priming. Cognitive Brain Research. 2005; 25: 589–606. 16202570 10.1016/j.cogbrainres.2005.08.011

[34] Curran T. Brain potentials of recollection and familiarity. Memory & Cognition. 2000; 28: 923–938. 11105518 10.3758/bf03209340

[35] Paller KA, Voss JL, Boehm SG. Validating neural correlates of familiarity. Trends in cognitive sciences. 2007; 11: 243–250. 17475539 10.1016/j.tics.2007.04.002

[36] Rayner K, Juhasz BJ, Brown SJ. Do readers obtain preview benefit from word N + 2? A test of serial attention shift versus distributed lexical processing models of eye movement control in reading. Journal of Experimental Psychology: Human Perception and Performance. 2007; 33: 230–245. 17311490 10.1037/0096-1523.33.1.230

[37] Drieghe D, Rayner K, Pollatsek A. Mislocated fixations can account for parafoveal-on-foveal effects in eye movements during reading. The Quarterly Journal of Experimental Psychology. 2008; 61: 1239–1249. 17853202 10.1080/17470210701467953 PMC2662923

[38] Rayner K. Eye movements in reading: Models and data. Journal of Eye Movement Research. 2009; 2: 1–10. 20664810 PMC2906818

