# Supplementary material for: Integration of Sentence-Level Semantic Information in Parafovea: Evidence from the RSVP-Flanker Paradigm
Source: PLoS One. 2015 Sep 29;10(9):e0139016. doi: 10.1371/journal.pone.0139016 (PMC4587981; doi:10.1371/journal.pone.0139016)
Supplement: S1 Appendix — (DOCX) [file pone.0139016.s001.docx]

Experimental Sentences:

**No Congruent Incongruent**

1 这帮人修筑了好几条路后受到了赞扬 这帮人解救了好几条路后受到了赞扬

2 农夫吃光儿子送来的饭后骑车走了 农夫晾干儿子送来的饭后骑车走了

3 美军炸掉了对方不少船希望速战速决 美军逮捕了对方不少船希望速战速决

4 孙飞飞养活了十来条蚕因而十分开心 孙飞飞织成了十来条蚕因而十分开心

5 小曾倒掉了那一碗水并拿来了辣椒 小曾捣碎了那一碗水并拿来了辣椒

6 李小萌切开了桌上那块饼并叫来了黄涛 李小萌修好了桌上那块饼并叫来了黄涛

7 老张调制出了西式的酒所以很兴奋 老张烧烤出了西式的酒所以很兴奋

8 父亲修剪了院子里的树后就上街了 父亲关掉了院子里的树后就上街了

9 杨华跨过地里那条沟时一点也不怕 杨华捉住地里那条沟时一点也不怕

10 总厂里冶炼的数百吨钢都被运往了分厂 总厂里纺织的数百吨钢都被运往了分厂

11 公司开采的大部分煤都被运往了北京 公司织成的大部分煤都被运往了北京

12 钱华力打中院里那些鸟后很兴奋 钱华力修平院里那些鸟后很兴奋

13 工人们建造了一座座桥以造福当地老百姓 工人们开垦了一座座桥以造福当地老百姓

14 民警没收了他所有的枪并逮捕了他 民警起诉了他所有的枪并逮捕了他

15 那群人拆除城西那座楼只用了半个月 那群人开垦城西那座楼只用了半个月

16 张凯奇烫伤了他的一只手后立马跑回家了 张凯奇扔掉了他的一只手后立马跑回家了

17 士兵们建造了好多的房给受灾的群众 士兵们搬运了好多的房给受灾的群众

18 李波料到了那一场雨但却没人相信他 李波出席了那一场雨但却没人相信他

19 宋晨割伤了陈元的脚可他不承认 宋晨偷走了陈元的脚可他不承认

20 徐叔叔出租了不少的屋给附近的学生 徐叔叔批发了不少的屋给附近的学生

21 徐丽帆擦拭了床头的灯然后才去洗澡 徐丽帆查阅了床头的灯然后才去洗澡

22 曹晓军捐献了好几次血因而受到了表扬 曹晓军擒获了好几次血因而受到了表扬

23 卓玛裁剪了很多的布以备以后用到 卓玛喂养了很多的布以备以后用到

24 黄小鹏冲洗了家里的车以迎接朋友 黄小鹏修剪了家里的车以迎接朋友

25 丹尼饲养了好几只羊想卖掉换钱 丹尼拆卸了好几只羊想卖掉换钱

26 厂里烧制的大多数砖被堆放在了外面 厂里纺织的大多数砖被堆放在了外面

27 红军招募了很多很多兵用来消灭敌军 红军制造了很多很多兵用来消灭敌军

28 许伯伯编成了十几张网想留给儿子 许伯伯复印了十几张网想留给儿子

29 韩梅摘掉了那几只瓜并拿来了袋子 韩梅捕获了那几只瓜并拿来了袋子

30 广场上积聚了很多很多人不知道是为什么 广场上整修了很多很多人不知道是为什么

31 同盟军轰炸了对方很多城可最后还是输了 同盟军活捉了对方很多城可最后还是输了

32 王大妈腌制屋子里的肉时非常地开心 王大妈清扫屋子里的肉时非常地开心

33 李阿姨扯裂了好几面旗以发泄情绪 李阿姨砸碎了好几面旗以发泄情绪

34 小赵铸造好了那把剑并送给了小李 小赵调试好了那把剑并送给了小李

35 徐峥冲洗了那几只碗后就出去了 徐峥放飞了那几只碗后就出去了

36 那伙人猎杀了那几只鹤并想将其运走 那伙人修补了那几只鹤并想将其运走

37 曹业书收起了自己那只鞋后显得很惆怅 曹业书划伤了自己那只鞋后显得很惆怅

38 王雪林冲洗了自己的头后就去玩了 王雪林修补了自己的头后就去玩了

39 宋子辰归还了李雷的书后什么也没说 宋子辰刺伤了李雷的书后什么也没说

40 陈若刷洗了那两只杯后看见了张俊 陈若宰杀了那两只杯后看见了张俊

41 吉姆观看了昨晚那场球并交了个朋友 吉姆出演了昨晚那场球并交了个朋友

42 王强国演唱了那几段戏后得到了称赞 王强国翻修了那几段戏后得到了称赞

43 小兔子跌入凳子旁边的洞后就看不到了 小兔子吃光凳子旁边的洞后就看不到了

44 李大伯烧制的那几把壶都非常漂亮 李大伯吹奏的那几把壶都非常漂亮

45 张莉莉做好了那几道菜后看见了父亲 张莉莉越过了那几道菜后看见了父亲

46 工厂开采了数千吨矿想拿来出口 工厂仿制了数千吨矿想拿来出口

47 刘阿姨清除了那一股味后就去院子了 刘阿姨割断了那一股味后就去院子了

48 孙晓扬起手里那把沙并挥了几下手 孙晓插入手里那把沙并挥了几下手

49 小约翰毒死了那几只虫后感觉很得意 小约翰修好了那几只虫后感觉很得意

50 李凌捉住后院那条蛇后喊来了父亲 李凌修平后院那条蛇后喊来了父亲

51 李琪张贴屋里那些画时突然打雷了 李琪配制屋里那些画时突然打雷了

52 郑军嵩移植了后院那些花后很开心 郑军嵩捕获了后院那些花后很开心

53 赵秋立倒掉了那几碗油并擦洗了桌子 赵秋立捣碎了那几碗油并擦洗了桌子

54 祝小明排练了那一支舞后显得很紧张 祝小明私藏了那一支舞后显得很紧张

55 人们填平了路两边的坑以防发生意外 人们栽种了路两边的坑以防发生意外

56 常刚伟演奏了很多的歌想用来赚生活费 常刚伟捡拾了很多的歌想用来赚生活费

57 田乐抄写了那几行字后去找李超了 田乐压倒了那几行字后去找李超了

58 吴品购买了那几支笔用来准备考试 吴品练习了那几支笔用来准备考试

59 李德超打碎了那几只瓶后偷偷进屋了 李德超放走了那几只瓶后偷偷进屋了

60 张利民拆开了门上的锁并清理了院落 张利民张贴了门上的锁并清理了院落

61 张健修理手里的那把伞大概用了十分钟 张健打磨手里的那把伞大概用了十分钟

62 郑海涛缝补了小时候的包因为舍不得扔 郑海涛装订了小时候的包因为舍不得扔

63 李岚添加了适量的醋后走出了屋子 李岚誊写了适量的醋后走出了屋子

64 王强一边摇动手里那把扇一边看小说 王强一边打磨手里那把扇一边看小说

65 张翠擦掉了裤子上的泥并洗了上衣 张翠缝补了裤子上的泥并洗了上衣

66 刘宇翔搬走了前院的碳并清理了杂物 刘宇翔摘掉了前院的碳并清理了杂物

67 何梦晓搬走了院里那些盆想腾出些地方 何梦晓拔掉了院里那些盆想腾出些地方

68 李靖荣点燃了那一堆柴后就回屋了 李靖荣痛骂了那一堆柴后就回屋了

69 林子涵摘掉了屋后那些梨并清扫了落叶 林子涵赶跑了屋后那些梨并清扫了落叶

70 那帮人猎杀了森林里的象后就消失了 那帮人采摘了森林里的象后就消失了

71 汪晴拆开了前院的箱并堆放好了它们 汪晴收割了前院的箱并堆放好了它们

72 刘辰昨晚吃光了柜子里的面但他却不承认 刘辰昨晚打碎了柜子里的面但他却不承认

73 刘雅切开刚刚买的糕并喊来了大家 刘雅试穿刚刚买的糕并喊来了大家

74 田丽丽拿走了餐桌上的茶并拿来了酒杯 田丽丽切开了餐桌上的茶并拿来了酒杯

75 曾杰购买的那两把铲昨天都坏掉了 曾杰吹奏的那两把铲昨天都坏掉了

76 何晨祥晾干了收来的谷并将它们装好了 何晨祥改造了收来的谷并将它们装好了

77 王荣清洗了新买的葱后就去休息了 王荣试穿了新买的葱后就去休息了

78 张桐铲除了路上的雪后得到了表扬 张桐填平了路上的雪后得到了表扬

79 赵小翠缝补了自己那双袜后叫来了姐姐 赵小翠冻伤了自己那双袜后叫来了姐姐

80 王达砸碎了篮子里的蛋后就不见了 王达赶跑了篮子里的蛋后就不见了

81 沈培擦掉了脸上的泪后出去了 沈培治愈了脸上的泪后出去了

82 黄主任治愈了刘航的眼后非常兴奋 黄主任出演了刘航的眼后非常兴奋

83 沈杰观看了那两盘碟后还不满足 沈杰吃光了那两盘碟后还不满足

84 王爱红加热了姐姐送的奶并拿来了点心 王爱红清洗了姐姐送的奶并拿来了点心
